# Association of the neoadjuvant chemotherapy cycle with survival outcomes in patients with locoregionally advanced nasopharyngeal carcinoma: a propensity-matched analysis

**DOI:** 10.18632/oncotarget.21587

**Published:** 2017-10-06

**Authors:** Wang Fangzheng, Jiang Chuner, Ye Zhimin, Sun Quanquan, Liu Tongxin, Xu Min, Wu Peng, Long Bin, Masoto Sakamoto, Wang Yuezhen, Yan Fengqin, Fu Zhenfu, Jiang Yangming

**Affiliations:** ^1^ Department of Radiation Oncology, Zhejiang Cancer Hospital, Zhejiang Hangzhou 310022, People’s Republic of China; ^2^ Zhejiang Key Laboratory of Radiation Oncology, Zhejiang Hangzhou 310022, People’s Republic of China; ^3^ Department of Radiology, Japanese Red Cross Fukui Hospital, Fukui 918-8501, Japan; ^4^ Department of Breast Tumor Surgery, Zhejiang Cancer Hospital, Zhejiang Hangzhou 310022, People’s Republic of China; ^5^ Department of Physics, Zhejiang Cancer Hospital, Zhejiang Hangzhou 310022, People’s Republic of China; ^6^ Department of Pathology, Zhejiang Cancer Hospital, Zhejiang Hangzhou 310022, People’s Republic of China; ^7^ Department of Nuclear Medicine, Zhejiang Cancer Hospital, Zhejiang Hangzhou 310022, People’s Republic of China; ^8^ Department of Didital Earth, Institute of Remote Sensing and Didital Earth, CAS, Beijing 100101, People’s Republic of China

**Keywords:** nasopharyngeal carcinoma, neoadjuvant chemotherapy, cycle, survival outcomes, prognosis

## Abstract

Neoadjuvant chemotherapy (NAC) is widely used to treat locoregionally advanced nasopharyngeal carcinoma (NPC). To determine the optimal number of NAC cycles, we assessed the effect of NAC cycle on survival outcomes of locoregionally advanced NPC patients receiving NAC before concurrent chemotherapy and intensity-modulated radiotherapy. Clinical data from 1,188 non-metastatic NPC patients were retrospectively reviewed. All received ≥2 cycles of NAC added to concurrent chemoradiotherapy. Propensity score matching (PSM) was used to identify paired patients according to various covariates. In total, 297 pairs were selected. After a median follow-up time of 57 months (range: 7 to 104 months), the 5-year locoregional relapse-free survival, distant metastasis-free survival (DMFS), progression-free survival (PFS), and overall survival rates in patients treated with 2 cycles vs. 3 to 4 cycles of NAC were 91.3% vs. 87.2% (*P*=0.149), 93.3% vs. 88.5% (*P*=0.043), 88.7% vs. 81.7% (*P*=0.037), and 94.0% vs. 92.6% (*P*=0.266), respectively. On multivariate analysis, 2 cycles of NAC were associated with improved DMFS (hazard ratio, 0.499; *P*=0.038) and PFS (hazard ratio, 0.585; *P*=0.049). NAC cycle was an independent prognosticator of DMFS and PFS in univariate and multivariate analyses. Thus, 2 cycles of NAC appear sufficient, as additional cycles were not associated with added survival benefit for locoregionally advanced NPC.

## INTRODUCTION

The incidence of nasopharyngeal carcinoma (NPC) is 15 to 50 cases per 100,000 annually in Southern China, Singapore, and Malaysia that vary with age, ethnicity, and geographical origin [1]. Radiotherapy (RT) is the standard treatment for NPC because of the anatomical location and the high radiosensitivity. Patients with locoregionally advanced NPC at diagnosis account for 60% to 70% of all NPC patients [2]. Intensity-modulated radiation therapy (IMRT) has been used to improve locoregional control but provides little benefit for survival outcome and prevention of distant failure [3, 4]. According to meta-analyses of randomized studies, combination RT and chemotherapy reduces the risk of mortality by 18% and increases 5-year survival by 4% to 6% [5]. Concurrent chemoradiotherapy (CCRT) with or without adjuvant chemotherapy, which provides a benefit in overall survival (OS), has become the standard treatment for locoregionally advanced NPC, although with acute toxicities [6–8]. A meta-analysis showed that compared with CCRT alone, addition of neoadjuvant chemotherapy (NAC) to CCRT reduces distant failure in locoregionally advanced NPC patients [9, 10], and another meta-analysis confirmed that NAC followed by CCRT improved progression-free survival (PFS) and OS [11]. However, the efficacy of the additional NAC to CCRT in patients with locoregionally advanced NPC remains controversial [12–14]. Sun et al. reported that 3 cycles of docetaxel, cisplatin, 5-fluorouracil-based induction *chemotherapy* (TPF IC) before CCRT improve survival outcomes, with 3-year OS of 92%, 3-year failure-free survival (FFS) of 80%, and 3-year distant metastases-free survival (DMFS) of 90% [15]. In the study by Kong et al. [16] of the TPF IC regimen in the treatment of locoregionally advanced NPC, 3-year OS, PFS, DMFS, and locoregional free-survival (LRFS) were 94.8%, 78.2%, 90.5%, and 93.9%, respectively [16]. Considering these results, addition of NAC to CCRT is a promising option for locoregionally advanced NPC patients in the era of IMRT.

However, the number of cycles of NAC that should be recommended for these patients is unclear. In previous studies [12–17], NPC patients received 2, 3, and even 4 cycles of NAC. More cycles of NAC delay IMRT delivery and prolong the wait time of IMRT. Unfortunately, longer IMRT wait time is associated with poor survival outcomes of NPC patients [18, 19]. Therefore, whether a threshold of NAC cycles exists, beyond which an impact on the survival of NPC patients is seen, needs further study. On the basis of this hypothesis, we performed a retrospective study to compare long-term survival outcomes of adding 2 cycles vs. 3 to 4 cycles of NAC to CCRT in locoregionally advanced NPC patients. To avoid the interference of covariates, we used the propensity score matching (PSM) methods to select paired patients [20].

## RESULTS

### Patient characteristics

The clinical data of 1,188 newly diagnosed locoregionally advanced NPC patients, who were initially treated with NAC followed by CCRT, were collected and retrospectively reviewed. From the original data, 297 pairs were selected by PSM. Basic characteristics of all patients are summarized in Table 1. For the selected subjects, the median age was 50 years (range, 18 to 77 years), and the ratio of males to females was 1.94:1 (392:202). There were no statistically significant differences in age, gender, stage, and treatment factors between 2 cycles and 3 to 4 cycles of NAC.

**Table 1:** Basic characteristic of 594 LA NPC patients between 2 cycles and 3 to 4 cycles of NAC

Characteristic	2 cycles of NAC	3 to 4 cycles of NAC	χ^2^	*P*
	No (%)	No (%)		
Sex			0.368	0.544
Male	200 (68.0)	192 (64.6)		
Female	97 (32.0)	105 (35.4)		
Age (years)			0.013	0.910
Range	19–77	18-74		
Median	49	50		
<60	252 (85.7)	250 ((84.2)		
≥ 60	45 (14.3)	47 (15.8)		
WHO pathology			0.739	0.691
I	6 (2.0)	4 (1.3)		
II	12 (4.0)	15 (5.1)		
III	278 (94.0)	277 (93.4)		
T stage^*^			1.772	0.621
T1	13 (4.4)	19 (6.4)		
T2	72 (24.2)	67 (22.6)		
T3	134 (45.1)	140 (47.1)		
T4	78 (26.3)	71 (23.9)		
N stage^*^			2.403	0.493
N0	29 (9.8)	21 (7.1)		
N1	71 (23.9)	67 (22.6)		
N2	143 (48.1)	159 (53.5)		
N3	54 (18.2)	50 (16.8)		
Clinical stage^*^			2.343	0.126
III	197 (66.3)	178 (59.9)		
IVA-B	100 (33.7)	119 (41.1)		
NAC regimen			1.473	0.689
TPF	83 (35.0)	85 (51.3)/(103)		
TP	104 (26.7)	92 (13.1)/(152)		
GP	57 (3.3)	58 (2.0)/(68)		
PF	53 (35.0)	62 (33.6)/(73)		
AC			146.081	<0.001
No	104 (35.3)	251 (84.5)		
Yes	193 (64.7)	48 (15.5)		
WRT (day)^#^				0.04
Range	30–104	49–206		
Median	49	82		

WHO, World Health Organization; AC, adjuvant chemotherapy; NAC, neoadjuvant chemotherapy; WRT, waiting time of radiotherapy.

^*^The 7th AJCC/UICC staging system. ^#^t-test.

### Survival outcomes

**Figure 1:**
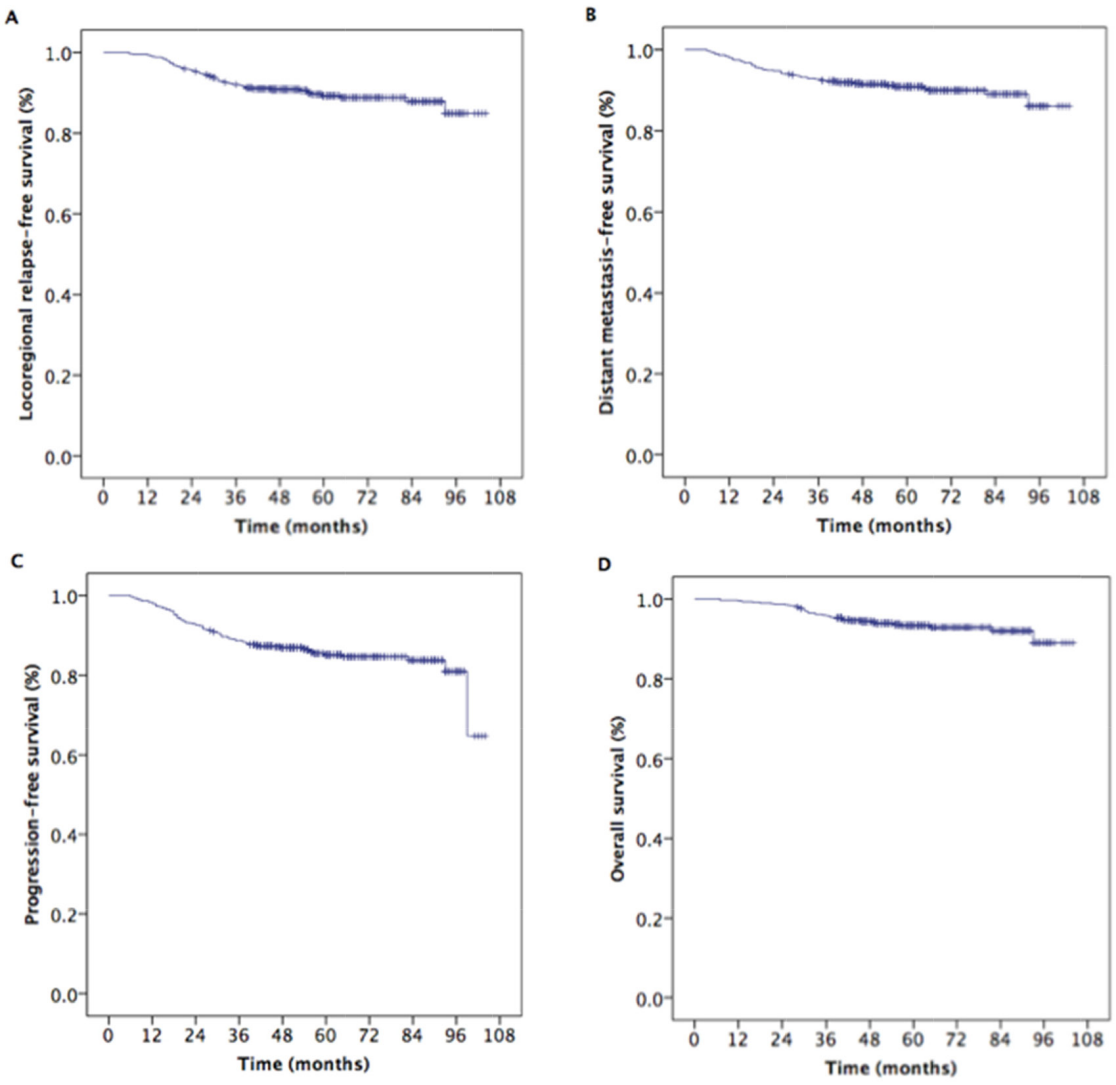
Kaplan-Meier estimates of the survival in 594 patients with nasopharyngeal carcinoma

With the median follow-up duration of 57 months (range, 7 to 104 months), the estimated 5-year locoregional relapse-free survival (LR-RFS), DMFS, PFS, and OS rates were 89.3%, 90.9%, 85.1%, and 93.3%, respectively (Figure 1).

**Figure 2:**
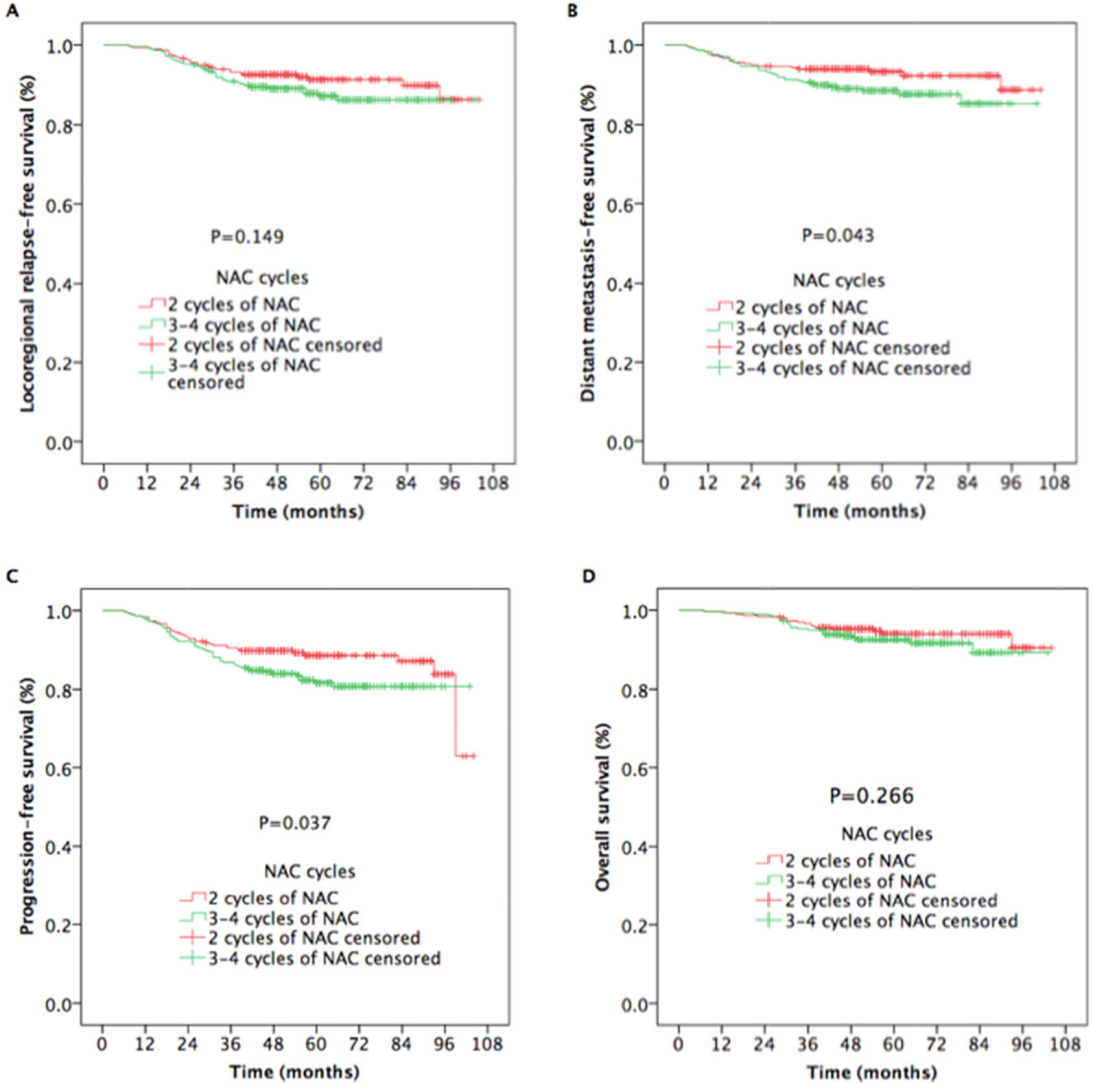
Kaplan-Meier estimates of the survival outcomes in nasopharyngeal carcinoma patients receiving 2 cycles and 3 to 4 cycles of NAC

The 5-year DMFS rate was higher for patients treated with 2 cycles of NAC than for those treated with 3 to 4 cycles of NAC (93.3% vs. 88.5%, respectively; *P* = 0.043) (Figure 2B). An improvement occurred in the 5-year PFS rate (88.7% vs. 81.7%, respectively; *P* = 0.037) (Figure 2C) in patients who received 2 cycles of NAC compared with those who received more cycles of NAC. A statistically significant difference in LR-RFS and OS was not found between the two groups (5-year LR-RFS: 91.3% vs. 87.2%, respectively, *P* = 0.149 [Figure 2A] and 5-year OS: 94.0% vs. 92.6%, respectively, *P* = 0.266 [Figure 2D]).

### Failure patterns

Treatment failure occurred in 88 patients (14.8%) by the last follow-up. In the 2-cycles group, 35 patients (11.8%) experienced “any” failure (locoregional relapse occurred in 14 patients, locoregional relapse and distant failure occurred in 12 patient, and distant metastases occurred in 9 patients), and 53 patients (17.8%) in the 3 to 4 cycles group experienced “any” failure (locoregional relapse occurred in 18 patients and locoregional relapse and distant failure occurred in 17 patients, and distant failure alone occurred in 17 patients). Patterns of treatment failure in NPC patients are listed in Table 2. Median time to failure in patients receiving 2 cycles of NAC versus 3 to 4 cycles of NAC was 22 months (range, 6 to 99 months) versus 26 months (range, 6 to 65 months), respectively.

**Table 2:** Treatment failure

Failure mode	2 cycles of NAC	3 to 4 cycles of NAC	*P*
	N = 297	N = 297	
Locoregional	14	18	0.218
Locoregional and distant	12	17	
Distant	9	17	
Nonfailure	262	245	

NAC, neoadjuvant chemotherapy.

### Identification of prognostic factors

The common potential prognostic factors included patient age, patient sex, clinical stage, adjusted tumor (T) and lymph node (N) stage, adjuvant chemotherapy (AC), NAC regimen, and NAC cycle. We identified which factors influenced survival outcome and evaluated the prognostic role of these factors by univariate and multivariate analyses. Univariate analysis showed that the 5-year DMFS, PFS, and OS of NPC patients with N0-N1 were superior to those of patients with N2-N3 (5-year DMFS: 97.3% vs. 88.0%, *P* = 0.001; PFS: 91.0% vs. 82.5%, *P* = 0.012; OS: 97.7% vs. 91.3%, *P* = 0.008), and the prognostic difference of DMFS and PFS was found between 2 cycles and 3 to 4 cycles of NAC (Table 3).

**Table 3:** Prognostic factors on survival outcomes of 594 NPC patients by use of univariate analysis

Characteristics	LRRFS (%)	*P*	DMFS (%)	*P*	PFS (%)	*P*	OS (%)	*P*
Sex		0.832		0.088		0.263		0.050
Male	89.3		89.1		84.0		91.7	
Female	89.1		94.6		87.5		96.7	
Age		0.164		0.824		0.737		0.061
<60	89.7		91.0		85.0		94.2	
≥60	86.9		90.2		85.8		88.5	
T stage		0.851		0.937		0.686		0.948
T1-T2	89.6		91.1		84.0		93.0	
T3-T4	89.2		90.9		85.4		93.8	
N stage		0.192		0.001		0.012		0.008
N0-N1	92.0		97.3		91.0		97.7	
N2-N3	88.4		88.0		82.5		91.3	
Clinical stage		0.111		0.004		0.016		0.008
III	90.5		93.5		87.3		95.6	
IVA/B	87.2		86.4		81.4		89.4	
AC		0.225		0.735		0.707		0.330
Yes	88.0		90.4		84.6		96.2	
No	91.6		91.6		86.1		92.7	
NAC regimen		0.450		0.850		0.662		0.945
TPF	88.2		91.6		83.9		93.6	
TP	91.7		91.2		87.5		92.5	
GP	93.8		91.8		89.8		95.9	
FP	86.2		88.5		82.6		92.7	
NAC cycle		0.149		0.043		0.037		0.266
2 cycles	91.3		93.3		88.7		94.0	
3 to 4 cycles	87.2		88.5		81.7		92.6	

LRRFS, locoregional relapse-free survival; DMFS, distant metastases-free survival; PFS, progression-free survival; OS, overall survival; AC, adjuvant chemotherapy; NAC, neoadjuvant chemotherapy; TP, docetaxel/cisplatin; TPF, docetaxel/cisplatin/fluorouracil; GP, gemcitabine/cisplatin; FP, cisplatin/fluorouracil.

Consistent with the results of univariate analysis, 2 cycles of NAC improved PFS (hazard ratio [HR], 0.585; 95% confidence interval [CI], 0.344–0.997; *P* = 0,049) and DMFS (HR, 0.499; 95% CI, 0.258–0.964; *P* = 0,038) (Table 4).

**Table 4:** Multivariate analysis of prognostic factors in 594 LA NPC patients

Endpoint	Variate	Category	HR	95% CI	*P*-value
OS	NAC cycle	2 cycles vs. 3 to 4 cycles	0.695	0.323–1.498	0.353
	Age	<60 vs. ≥60 years	0.451	0.209–0.973	0.042
	N stage^*^	N0-N1 vs. N2-N3	0.272	0.105–0.707	0.008
PFS	NAC cycle	2 cycles vs. 3 to 4 cycles	0.585	0.344–0.997	0.049
	N stage^*^	N0-N1 vs. N2-N3	0.483	0.280-0.834	0.009
LRRFS	NAC cycle	2 cycles vs. 3 to 4 cycles	0.820	0.459-1.532	0.535
DMFS	NAC cycle	2 cycles vs. 3 to 4 cycles	0.499	0.258–0.964	0.038
	N stage^*^	N0-N1 vs. N2-N3	0.239	0.101–0.565	0.001

OS, overall survival; PFS, progression-free survival; LRRFS, locoregional recurrence-free survival; DMFS, distant metastasis-free survival; NAC, neoadjuvant chemotherapy.

^*^The 7th AJCC/UICC staging system.

### Subgroup analysis

**Figure 3:**
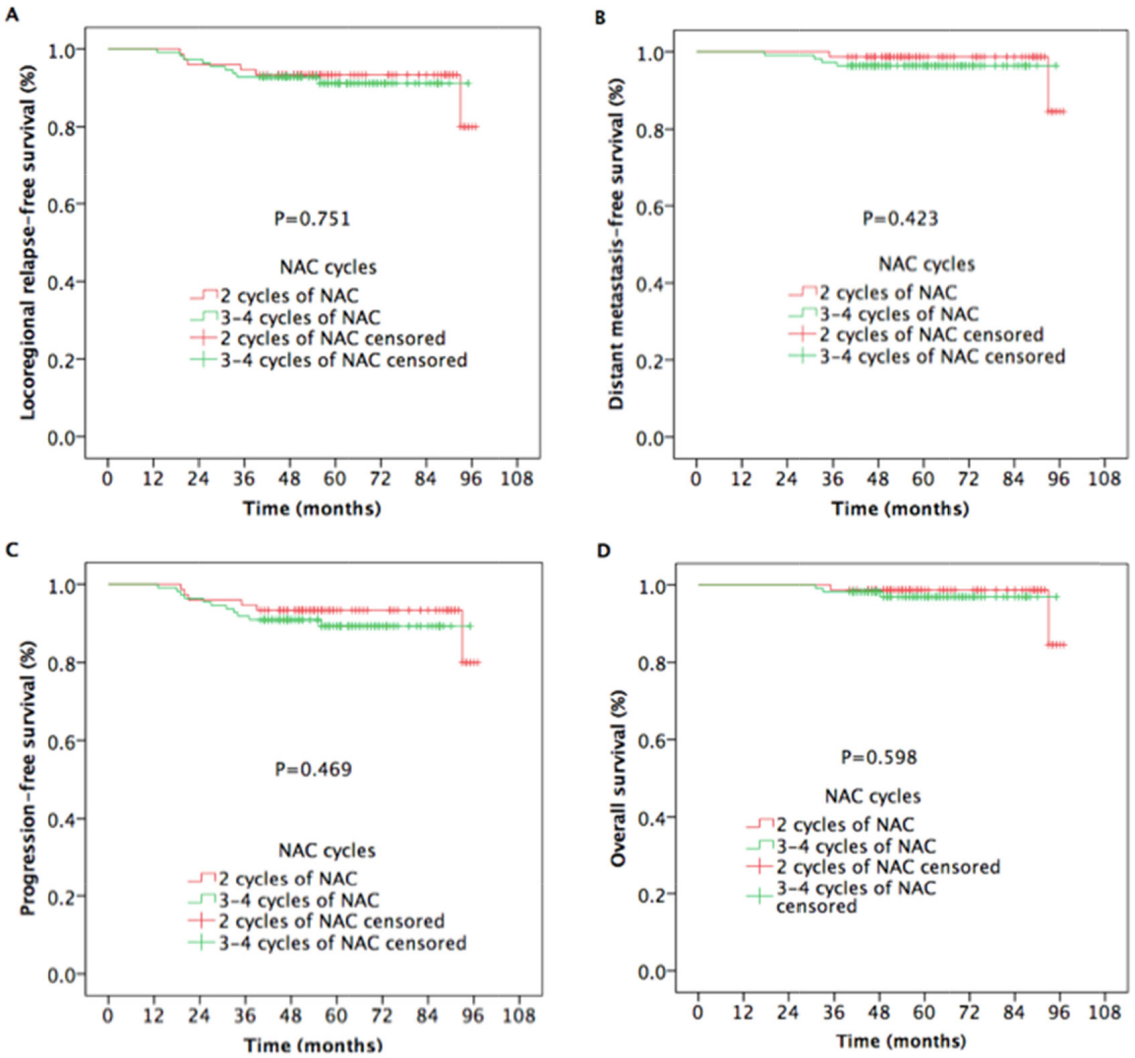
Kaplan-Meier estimates of survival outcomes in nasopharyngeal carcinoma patients with N0-N1 receiving 2 cycles and 3 to 4 cycles of NAC

We found that N category was an independent negative prognostic factor. Moreover, 5-year survival outcomes in NPC patients with N0-N1 were superior to those in patients with N2-N3. Therefore, we preformed the subgroup analysis to assess the prognostic value of NAC cycle in NPC patients according to N category. In NPC patients in the N0-N1 category, the 5-year LRRFS (93.3% vs. 91.1%, respectively; *P* = 0.751[Figure 3A]), DMFS (98.7% vs. 96.4%, respectively; *P* = 0.423 [Figure 3B]), PFS (93.3% vs. 89.3%, respectively; *P* = 0.469[Figure 3C]), and OS (98.7% vs. 96.9%, respectively; *P* = 0.598 [Figure 3D]) were comparable between 2 cycles and 3 to 4 cycles of NAC.

**Figure 4:**
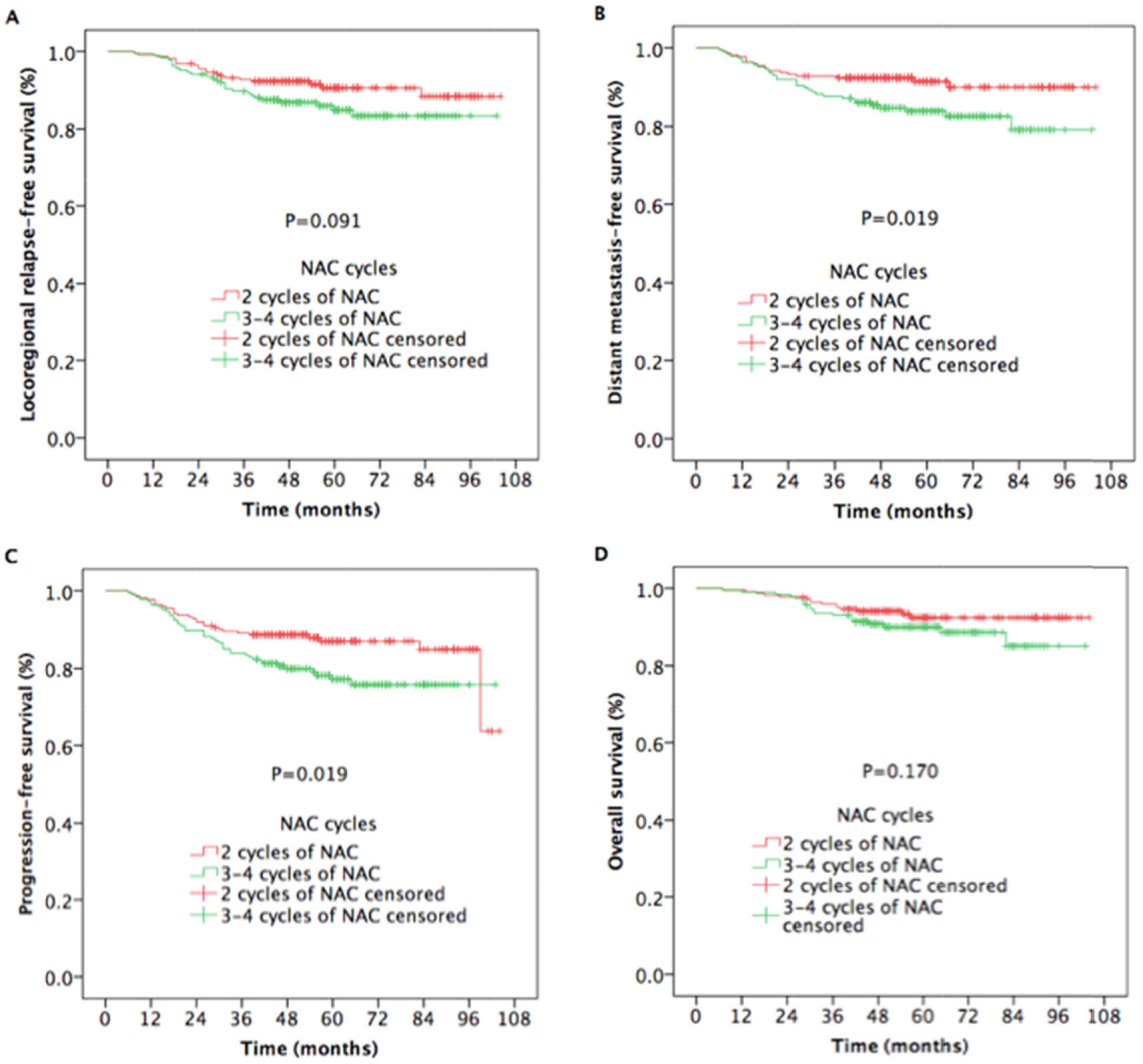
Kaplan-Meier estimates of survival outcomes in N2-N3 nasopharyngeal carcinoma patients receiving 2 cycles and 3 to 4 cycles of NAC

Likewise, for patients in the N2-N3 category, the 5-year DMFS (91.4% vs. 83.9%, respectively; *P* = 0.019 [Figure 4B]) and PFS (87.0% vs. 77.2%, respectively; *P* = 0.019 [Figure 4C]) rates in patients receiving 2 cycles of NAC were higher than those in patients receiving 3 to 4 cycles of NAC, and the differences of 5-year LRRFS (90.6% vs. 84.9%, respectively; *P* = 0.091[Figure 4A]) and OS (92.4% vs. 90.0%, respectively; *P* = 0.170 [Figure 4D]) between the two groups did not reach statistical significance.

## DISCUSSION

This study revealed the impact of NAC cycle on survival outcomes in NPC patients by use of PSM and long follow-up time, and the results showed that 5-year DMFS and PFS in NPC patients receiving 2 cycles of NAC were higher than in NPC patients receiving 3 to 4 cycles of NAC. In addition, subgroup analysis demonstrated that adding more than 2 cycles of NAC to CCRT was associated with poor DMFS and PFS. Accordingly, we can draw a conclusion that 2 cycles of NAC might be enough and additional cycles are not associated with survival benefit for locoregionally advanced NPC.

A randomized phase III study showed that 3 cycles of TPF IC before CCRT improve survival outcomes, with 3-year OS of 92%, 3-year FFS of 80%, and 3-year DMFS of 90% [15]. In a randomized phase II trial by Hui et al. [21], 2 cycles of TP IC before CRT improved 3-year OS compared with CRT alone (94.1% vs. 67.7%, *P* = 0.0112). Kong et al. [16] reported that the 3-year PFS, DMFS, LRFS, and OS in patients with N3 disease were 81.8%, 81.8%, 100%, and 90.9%, respectively, after 4 cycles of NAC before CCRT. Thus, 2 cycles to 4 cycles of NAC have been widely used in clinical practice for locoregionally advanced NPC and improved survival outcomes of these patients. More NAC cycles could prolong the wait time of IMRT. In the current study, the median wait times were 49 days and 82 days, respectively, and a statistically significant difference was seen between 2 cycles and 3 to 4 cycles of NAC (*P* = 0.040).

The wait time of IMRT was the interval time between cancer diagnosis and radical IMRT. Generally, cancer patients should receive radical treatment as early as possible after they are diagnosed. However, many patients were hindered in receiving timely treatment because of social factors, limited medical resources, scarcity of policy support, and treatment-related factors [22]. Unfortunately, a correlation between long wait time and poor survival outcome was found in many cancers, including breast cancer, rectal cancer, bladder cancer, and head-and-neck cancer [23–26]. Chen et al. [18] showed that a longer wait time beyond 4 weeks was associated with worse PFS, but not worse OS, in 814 NPC patients with a short-term follow-up. Another study with a large population and long follow-up revealed a correlation between prolonged wait times more than 30 days and poor survival outcome [19]. From the above two studies, we determined the influence of wait time on the survival outcome for NPC. NAC is a primary factor in longer wait time even though it has been shown to improve survival outcomes in locoregionally advanced NPC. The wait time for patients who received ≥2 cycles of NAC was more than 30 days. Thus, longer wait time reduces survival benefits of NAC in patients with locoregionally advanced NPC. So, we recommend ≥2 cycles of NAC as optimal before timely IMRT for locoregionally advanced NPC. A study by Peng et al. [27], with 247 pairs of NPC patients, including limited clinical characteristics, found no significantly prognostic difference between 2 cycles and >2 cycles of NAC, but stratified analysis demonstrated that patients receiving 2 cycles of NAC had better OS than patients receiving >2 cycles, and NAC was an independent prognostic factor of OS for the N2-N3 category. However, in the study, the wait time was not tested between the two groups. Consequently, >2 cycles of NAC were not proved to be associated with worse survival outcomes, although a statistically significant difference for N2-N3 between the two groups was seen.

In the current study, on the basis of long follow-up times, 5-year LRRFS, DMFS, PFS, and OS rates between two groups were 91.3%, 93.3%, 88.7%, and 94.0% and 87.2%, 88.5%, 81.7%, and 92.6%, respectively. However, statistically significant differences in DMFS and PFS were seen between the two groups. We identified which factors influenced survival outcome and evaluated the prognostic role of these factors by univariate and multivariate analyses. We found that N category was an independent prognostic factor of LRRFS, DMFS, PFS, and OS, and NAC cycle was an independent predictor of DMFS and PFS.

We used PSM and multivariate analysis to assess the prognostic role of NAC cycle for locoregionally advanced NPC. Our study has some limitations because of the retrospective nature, single center, and heterogeneous NAC regimens. Further prospective trails are warranted.

We found that >2 cycles of NAC before CCRT resulted in better DMFS and PFS in patients with locoregionally advanced NPC. However, because of the retrospective nature of the study, our results should be regarded as preliminary.

## MATERIALS AND METHODS

### Patients

The patients enrolled in this study were hospitalized from May 2008 to April 2014 in the Department of Radiation Oncology, Zhejiang Cancer Hospital. The eligible patients met the following criteria: (i) histologically proved locoregionally advanced NPC, (ii) Eastern Cooperative Oncology Group performance status ≤ 1, (iii) completion of radical IMRT, (iv) received 2 to 4 cycles of NAC before CCRT, and (v) received no previous anti-cancer treatment. Of 1,188 patients, 594 (50.0%) were matched for the current study. This retrospective study was approved by the Medical Ethics Committee of Zhejiang Cancer Hospital. All the patients signed informed consent forms.

### Baseline examinations

Patients had pretreatment evaluations that included complete histories, physical examinations, hematology and biochemistry profiles, chest radiographs, sonography of the abdomen, bone scans, magnetic response images of the nasopharynx, and nasopharyngoscopies. All patients were staged according to the 2010 American Joint Committee on Cancer staging system. Tumor histology was classified per the World Health Organization classification.

### Intensity-modulated radiotherapy

All patients underwent radical IMRT with simultaneous integrated boost technique that utilized 6-MV photons 2 to 3 weeks after IC. The delineation of target volumes of NPC during the treatment of IMRT was described previously [28–31]. Gross tumor volumes (GTVs) of primary tumor and the metastatic lymph nodes were defined as GTVnx and GTVnd, which were delineated according to pre-IC and post-IC MR images, respectively. The clinical target volume of nasopharynx (CTVnx) was defined as GTVnx plus a 7-mm margin that encompassed the nasopharyngeal mucosa plus 5-mm submucosal volume. The high-risk clinical target volume (CTV1) included the entire nasopharyngeal cavity, the anterior one-third to two-thirds of the clivus, the skull base, the pterygoid plates, the parapharyngeal space, the inferior sphenoid sinus, the posterior one-quarter to one-third of the nasal cavity, and the maxillary sinus and any lymph nodes in drainage pathways containing metastatic lymph nodes. The low-risk clinical target volume (CTV2) included levels IV and Vb without metastatic cervical lymph nodes.

The PTV was constructed automatically on the basis of each volume with an additional 3-mm margin in three dimensions to account for set-up variability. All the PTVs, including PGTVnx, PTVnx, PTV1, and PTV2, were not delineated outside of the skin surface. Critical normal structures, including the brainstem, spinal cord, parotid glands, optic nerves, chiasm, lens, eyeballs, temporal lobes, temporomandibular joints, mandible, and hypophysis were contoured and set as OARs during optimization.

The prescribed radiation dose was 69 or 72 Gy to PGTVnx, 66-70 Gy to PGTVnd, 62 to 66 Gy to PTVnx, 60 to 63 Gy to PTV1, and 51 to 54 Gy to PTV2, delivered in 30 or 33 fractions. Radiation was delivered once daily, five fractions per week, over 6 to 6.5 weeks for IMRT planning. The dose to OAR was limited on the basis of the RTOG 0225 protocol.

### Chemotherapy

All patients were given 2 to 4 cycles of platinum-based induction chemotherapy every 3 weeks. The available NAC regimens included TPF (docetaxel 60 mg/m^2^/d on day 1, cisplatin 25 mg/m^2^/d on days 1 to 3, and 5-fluorouracil 500 mg/m^2^/d on days 1 to 3), TP (docetaxel 60 mg/m^2^/d on day 1 and cisplatin 25 mg/m^2^/d on days 1 to 3), GP (gemcitabine 1,000 mg/m^2^/d on days 1 and 8 and cisplatin 25 mg/m^2^/d on days 1 to 3), and FP (cisplatin 25 mg/m^2^/d on days 1 to 3 and 5-fluorouracil 500 mg/m^2^/d on days 1 to 3).

The patients in this study underwent concurrent chemotherapy with cisplatin (80 mg /m^2^) divided into 3 doses administered in 3 days every 3 weeks for 2 to 3 cycles and received 21-day cycles of adjuvant chemotherapy with FP (cisplatin 25 mg/m^2^/d on days 1 to 3, and 5-fluorouracil 500 mg/m^2^/d on days 1to 3) or GP regimens within 3 to 4 weeks after RT.

### Efficacy evaluation and follow-up

The assessment of tumor response was performed three times after the completion of IC, at the end of IMRT, and 3 months after irradiation, which was based on MRI and nasopharynx fiberscope per the Response Evaluation Criteria for Solid Tumors. Systemic chemotherapy adverse events were graded per the National Cancer Institute Common Toxicity Criteria (NCI CTCAE Version 3.0), and RT-induced toxicities were scored per the Acute and Late Radiation Morbidity Scoring Criteria of the Radiation Therapy Oncology Group.

All the subjects underwent weekly examinations for treatment response and toxicities during RT. Patients were followed every 3 months in the first 2 years, every 6 months from the third to the fifth year, and then annually. Each follow-up included careful examination of the nasopharynx and neck nodes by an experienced doctor. MRI scans of the nasopharynx, nasopharynx fiberscope, chest computed tomography radiograph, and ultrasound of abdomen were performed 3 months after the completion of RT and every 6 to 12 months thereafter. Additional examinations were performed when indicated to evaluate local relapse or distant metastasis.

### Statistical analysis

The end points of this study included LRRFS, DMFS, PFS, OS, and acute toxicities from IC and CCRT. OS was calculated from the date of enrollment in the trail to the date of death or the last follow-up. LRRFS, DMFS, and PFS were calculated from the date of enrollment in the trail to the date of locoregional relapse, distant metastasis occurrence and the diagnosed evidence of disease progression, or the last follow-up, respectively. After recurrence or metastasis, patients were given salvage therapy as determined by their physicians.

Descriptive statistics were used to compare the patients’ characteristics and patterns of failure between the two arms. Two independent sample non-parametric tests were used to compare the acute toxicity between the two arms. Survival curves were generated by application of the Kaplan-Meier method. The curves were compared by use of log-rank tests. Multivariate analysis was performed by use of Cox regression models to identify significant prognostic factors. HRs and 95% CIs were calculated for each prognostic factor. IBM SPSS Statistics Version 19.0 was used for all data analysis. A *P* < 0.05 was considered statistically significant.

## CONCLUSION

We observed that an additional 2 cycles of NAC followed by CCRT might be enough and adding more cycles is not associated with survival benefit in locoregionally advanced NPC patients. Further randomized, controlled, multicenter phase III clinical trials are needed to confirm the therapeutic gain.
